# The Effects of NBF Gingival Gel Application in the Treatment of the Erosive Lichen Planus: Case Report

**DOI:** 10.3889/oamjms.2016.026

**Published:** 2016-01-29

**Authors:** Mirjana Popovska, Jasmin Fidovski, Sonja Mindova, Katerina Dirjanska, Stevica Ristoska, Emilija Stefanovska, Vera Radojkova-Nikolovska, Kristina Mitic, Biljana Rusevska

**Affiliations:** 1 *Faculty of Dentistry, Ss. Cyril and Methodius University of Skopje, 1000 Skopje, Republic of Macedonia*; 2 *PHO „Fjolla Medika”, Skopje, Republic of Macedonia*

**Keywords:** erosive lichen planus, therapy, topical treatment, NBF gingival gel

## Abstract

The therapy of erosive lichen planus (ELP) has been particular problem in the treatment of oral lesions. This case of ELP in male patient 29 years old was treated with topic application of the NBF gingival gel, three times a day after meal, previously rinsed with Clorhexidine gluconate 0.12%. After 5 days of treatment, initial improvements were recorded, and after two weeks of application of the NBF gingival gel we observed significant improvement. Clinical monitoring after the fifth day showed mild epithelialization of the eroded mucosa, yet still present erythematous base of the lesion. After the second week the erythema area was significantly reduced and the eroded surfaces of the mucosa were minimal, measured less than 0.5 mm. After the third week there were no erosions to detect on the oral mucosa, yet still present vague redness, which completely pulled after the fourth week. Treatment ended after the fifth week when the topical application of the NBF gingival gel was terminated, and therapy was done, and clinically achieved effects remained stable even after the third month of the treatment. Topic application of the NBF gingival gel with ELP patients showed positive clinical effects in relatively short time period.

## Introduction

Oral lichen planus (OLP) is a chronic disease, with unknown etiology, where the autoimmune mechanisms take a particular place. It is believed that the increased production on free radicals (ROS), may play an important role in the pathogenesis of various skin diseases, such as atopic dermatitis, psoriasis, vitiligo [1], and lichen planus, where the presence of oxidative stress is recently been proved [2]. Actually, the oxidative stress is caused between theimbalance in production of reactive free radicals (ROS) and the organism’s ability to neutralize reactive intermediaries, which are the consequence of the caused impairment [3]. Created lesions with potentially-present comparative DNA, result of the oxidative stress, can contribute to the development of the malignancy in the oropharyngeal area [4, 5]. It happens at lichen planus atrophic, bullous and erosive type, but usually, not at reticular type.

If we are called on the present oxidative stress, it is completely logical, that the disease will be treated with topical antioxidants, which on some level are our therapeutic experience.

Although the findings suggest that corticosteroids are the number one cure of ELP even when they are applied topically, though it is likely that certain patients can not apply them because of the numerous long-term effects. From this follows the need for new preparations to treat ELP.

In the last years, in the treatment of oral lesions advantages belong to the natural products containing propolys. This bee product is a rigid resin containing wax and herbal extracts used in the prevention of oral disease and has no side effects [6, 7]. Besides numerous anti-inflammatory properties [8] also it has antioxidants [9].

Antioxidants control oxidative stress in wound healing and there is a belief that it can accelerate the process of healing. Antioxidant therapy is in its beginning and is the most modern approach in the treatment of oral lesions. Propolys can be a component of tooth paste, water for washing, and gels and it can be easily applied to the oral cavity [10].

In dentistry it is widely used, and the findings are the subject of several studies. It is used for reducing dentinal hypersensitivity [11], prevents caries [12], affects the improvement of oral mucositis [13], and it was found that it could be useful for oral cancer [14].

Products based on nanotechnology, which contain antioxidants, may be excellent adjuvants in the healing of lesions. The only such product in the world is NBF gingival gel, which contains propolys, vitamin C and vitamin E in form of nanoemulsion.

This paper presents the case of oral erosive lichen planus localized in the buccal mucosa in a young male patient, which is topically applied NBF gingival gel and monitored for three months.

## Case Report

Male patient, 29 year old, was accepted to the University Dental Clinic Center, St. Pantelejmon” in Skopje, at the Department for oral medicine and periodontology, with basically complaints of discomfort, a sense of burning pain that intensifies during eating. Patient complains on serious problems in maintaining everyday oral hygiene.

**Figure 1:**
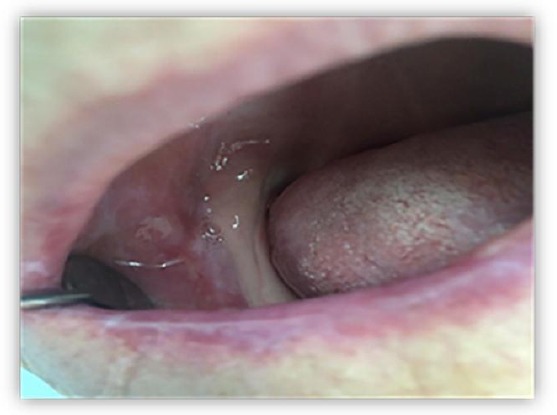
*Erosive lichen planus in the retro molar right buccal mucosa*.

Clinical investigating of buccal mucosa was presented with erosions superficial ulceration, erythematous background, Wickham stretch marks and solitary papules. Changes were localized unilaterally in retro molar region easily extended to occlusal line with dimensions of 1 mm in width and 1.5 mm in length on buccal mucosa on the right side (Fig. 1).

Clinical findings and history of the disease in addition of lichen planus were suspicious, so we applied additional criteria. For the definition of the diagnosis were implemented following procedures: biopsy for histopathological verification and direct immunofluorescence. Histopathological researches of buccal mucosa confirmed: canceled epithelium, degenerative changes in basal layer, and the individual basal cells atrophic-term changes.

**Figure 2:**
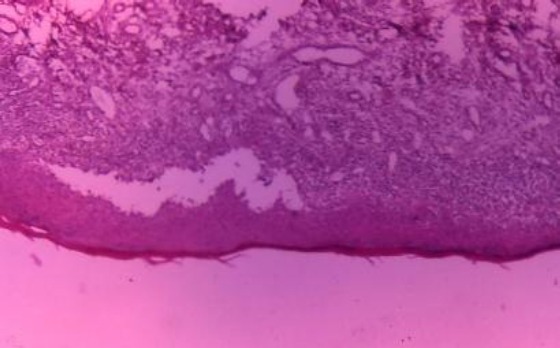
*Histopathological findings at erosive lichen planus*.

The surface of the epithelium was canceled with signs of erosion or deeper ulcers, around which was presented expressed cellular infiltration. In papilar layer was recorded intense lympho-histiocytes infiltration, which extend in strips. The present infiltrate has touched the epithelium and destroy the relation among the epithelium and corium (Fig. 2).

**Figure 3:**
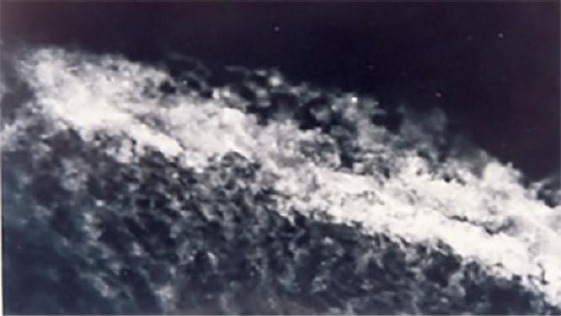
*Deposits of immunoglobulin G by the length of the epithelial basement membrane and superficial layer of the lamina propria at oral lichen planus*.

**Figure 4:**
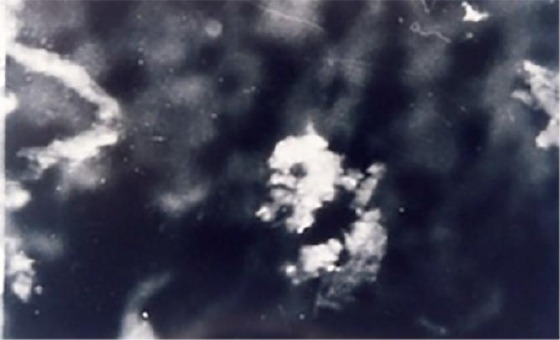
*Deposits of immunoglobulin C3 by the length of the epithelial basement membrane and superficial layer of the lamina propria at oral lichen planus*.

The direct immunofluorescence displayed an easy deposit of IgG and C3 component of complement at length on epithelial basement membrane (Figure 3 and Figure 4).

After 3 months of tissue biopsy, buccal mucosa resulted with completely epithelialization. After 2 months, and fully satisfactory findings, patient came again with the same subjective complaints as 5 month earlier and clinical findings presented with erosion which lie on the erythema surface of right retromollar area of buccal mucosa. Patient suggests to begin with topical use of the NBF gingival gel three times a day after eating (after they get a check, manually check dinners), with previous rinsing with Clorhexidine gluconate 0.12 %.

The patient was suggested not to receive food or drinks in the next 20 minutes, and gel to be protected by sterile gauze. Also we administered Nystatin, 4 times per 25 drops orally, due to prevention of candidiasis.

**Figure 5:**
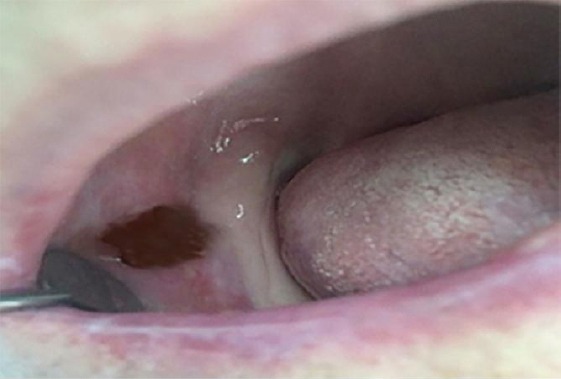
*Application of the NBF gingival gel at the site of the buccal mucosae lesion*.

Systemic application of corticosteroids at this moment was avoided (Figure 5).

The patient was followed continuously from the beginning until the end of the treatment.

The first three weeks controls were conducted three times a week, and after the fourth week, once a week in the following three months.

After 5 days of treatment, initial improvements were recorded, and after two weeks of application of the NBF gingival gel we observed significant improvement. Clinical monitoring after the fifth day showed mild epithelialization of the eroded mucosa, yet still present erythematous base of the lesion.

After the second week the erythema area was significantly reduced and the eroded surfaces of the mucosa were minimal, measured less than 0.5 mm.

**Figure 6:**
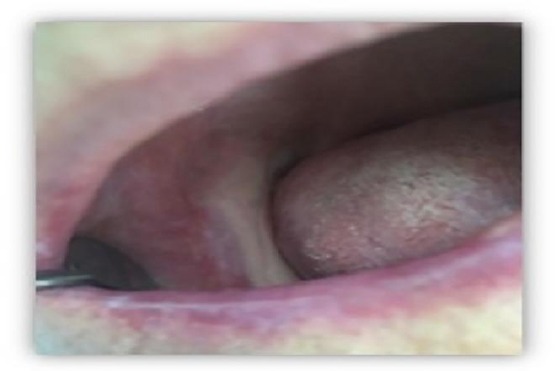
*Healed mucosal lesion after third week of therapy with NLB gingival gel*.

After the third week there were no lesions to detect on the oral mucosa, yet still present slight redness, which completely pulled after the fourth week. Treatment ended after the fifth week when the topical application of the NBF gingival gel was terminated, and therapy was done (Figure 6).

The results remained unchanged after the third month. Patients had no symptoms, and the clinical findings were satisfactory with no longer presence of erosions or erythema. Only present were Wickham stretch marks as whitish strings in stripe layout.

## Discussion

The main cause in the therapy of ELP is complete withdrawal of patients’ painful symptoms, epithelialization of the eroded mucosal lesions and oral carcinoma prevention. There are various therapeutic procedures described and implemented in practice till this day including topical medications, steroid ointments, ultraviolet rays (PUVA) [15] and laser [16]. However, the era of new medications increasingly conquers the world, and is also applied in the treatment of the oral mucosal lesions, including ELP. Various drugs are used either as topically applied or systemically applied medications [17].

In this field in the last 10 years there has been considerable development in the nanotechnology. Nanotechnology brought mankind in completely new period of its development where we have new therapeutic options in which central spot belongs to the nano materials. They contain particles that can be resorbed quickly and transported to the eroded tissues, where they stimulate healing, which is result of numerous biochemical and cellular interactions [18]. In the recovery process priority belongs to the antioxidants. With their use, the oxidative stress in the wound healing process is effectively controlled and probably due to them the whole healing can be accelerated. For now, the use of antioxidant therapy in the treatment of ELP is still in its infancy and is the newest and most modern therapeutic approach.

The products which are based on nanotechnology and contain antioxidants can be used as excellent adjuvants in the healing of oral mucosal lesions. Such product in the world is NBF gingival gel, containing vitamin C, vitamin E and propolis in form of nano emulsion.

In this case report where recovery process has been followed there had been significant solid therapeutic effects after topical application of the NBF gingival gel. Reduced painful symptomatology, facilitated mastication and complete epithelialization in period of four weeks are accounted as quite satisfactory findings. Easily accessible lesions can be treated by applying adherent pastes. They allow long-term retention of the paste on the site of the lesion, easy and precise control of the therapeutic effects of its application [19].

The main advantages of the topical application is that it provides reduced side effects, efficient delivery of small amounts of the drug in the affected area, without the possibility the drug to be lost in vain, distributed throughout the whole organism, simultaneously with maximizing in the affected area [20].

Essentially, pathological diagnosis of all parts of the body including those of the oral mucosa is the consequence of the oxidative stress which is an imbalance between the oxidants and antioxidants. This imbalance results in increased production of free radicals, including the reactive free radicals (ROS). They easily damage the oral cells, because the mucous membrane allows rapid absorption of the substances through their surfaces.

ROS and antioxidant imbalance can play leading role in the development of ELP [21, 22]. In fact, antioxidant defense mechanisms are very complex, and its debatable point is in the estimate amount and effects of the various antioxidants in vivo in assessment in the overall antioxidant status [21, 23].

Relevant data indicate that the role of the nutrients, especially of those so-called antioxidants as vitamin A, vitamin C and vitamin E is great significance in the pathogenesis of the ELP [24].

In the study of Abdolsamadi [25] level of anti-oxidants A, C and E in the saliva were significant lower in patients with OLP than healthy control group. The possible protective effects of vitamins in reducing the risk of OLP may be related to anti-oxidant role in eliminating free radical damage and single oxygen. The fact that antioxidant vitamins are effective against oxidative stress may explain the significant difference in oral mucosa [26, 27].

NBF gingival gel is high functional nano-bio fusion gel, created for the first time, with a new technology named nano-bio fusion. The gel contains three bio-compatible nano-emulsion contents (Vitamin C, Vitamin E, and Propolis extract) which have antibacterial, ant inflammatory and anti-oxidative effect. Vitamin C and Vitamin E have power anti-oxidant effect and Propolis which content flavonoids.

According to well-known effect of anti-oxidants, in this case report we used nano emulsion in treatment of ELP at different periods of time. The obtained results were satisfied.

The first improvement was evident after the fifth day with less epithelization. After two weeks, it was evident more epithelization with erithema reduction. Re-epithelization process reaches a maximum after three weeks, without erithema. We believe that all effects are due to Vitamin C, Vitamin E, and Propolis.

Vitamin C is known to activate phagocytes, and have the primary role in organism defense [28].

It‘s a co-factor for about eight enzymatic reaction in the synthesis of collagen. Vitamin C is shown to accelerate gingival wound healing in experimental animals, preventing the bleeding [29, 30]. The molecule of nano Vitamin C that is present in the gel, is 2 times smaller, and 110 times more potent in the synthesis of collagen, than vitamin C on its own. So, its elimination can be realized faster, easier, and more efficient.

Anti-oxidant property of propolis was evaluated by Krol [31], biocompatibility by Bankova [32], and antibacterial, antifungal, ant oxidative and anti-inflammatory properties by Sforcin [33].

One of the important components which contribute to the achieved result following topical application of NBF gingival gel is adhesiveness. Its constitutive component – propolis ensures very good adhesion, while the nanoemulsion itself has a much smaller surface tension due to the small dimensions of the vitamin molecules, being the reason for the instantaneously rapid absorption through the mucose in the target cells. Furthermore, the main component of the propolis - flavonoids adds to the antibacterial, antifungal, antiviral, anti-oxidative and anti-inflammatory properties.

Without limitations, and within the findings of this case report, it can be concluded that topic application of NBF gingival gel at ELP, showed positive clinical effects in a relatively short period of time, thus avoiding application of systematic or topical steroids due to their numerous adverse effects.

NBF gingival gel, with its own therapeutic modalities, allows us to recommended in the treatment of OLP.
